# Adams-Stokes attack as the first symptom of acute rheumatic fever: report of an adolescent case and review of the literature

**DOI:** 10.1186/1824-7288-38-61

**Published:** 2012-10-30

**Authors:** Nicola Carano, Ilaria Bo, Bertrand Tchana, Erica Vecchione, Silvia Fantoni, Aldo Agnetti

**Affiliations:** 1 Department of Paediatrics – Paediatric Cardiology Unit, University of Parma, via Gramsci, 14 43126, Parma, Italy; 2 Post – Graduate School of Paediatrics - Department of Paediatrics, University of Parma, via Gramsci, 14 43126, Parma, Italy

**Keywords:** Adams Stokes, Complete heart block, Rheumatic fever

## Abstract

**Background:**

Acquired complete heart block, in pediatric age is mainly the results of direct injury to conduction tissue during cardiac surgery or cardiac catheterisation. It can also be observed in different clinical settings as infectious diseases, neoplasia, and inflammatory diseases. It has a wide range of presentation and in some settings it can appear a dramatic event. Although a rare finding during acute rheumatic fever, with a transient course, it may need a specific and intensive treatment.

**Case presentation:**

We report the case of an Adams-Stokes attack in an adolescent with acute rheumatic carditis and complete atrio-ventricular block. The attack was the first symptom of carditis.

We reviewed the literature and could find 25 cases of complete atrio-ventricular block due to rheumatic fever. Ten of the 25 patients experienced an Adams-Stokes attack. Nineteen of the 25 patients were certainly in the pediatric age group. Seven of the 19 pediatric cases experienced an Adams-Stokes attack. In 16/25 cases, the duration of the atrio-ventricular block was reported: it lasted from a few minutes to ten days. Pacemaker implantation was necessary in 7 cases.

**Conclusion:**

Rheumatic fever must be kept in mind in the diagnostic work-up of patients with acquired complete atrio-ventricular block, particularly when it occurs in pediatric patients. The insertion of a temporary pacemaker should be considered when complete atrio-ventricular block determines Adams-Stokes attacks. Complete heart block during acute rheumatic fever is rare and is usually transient. Along with endocarditis, myocarditis and pericarditis, complete atrio-ventricular block has been recognized, rarely, during the course of acute rheumatic carditis.

## Background

Acquired complete heart block, in paediatric age is mainly the results of direct injury to conduction tissue during cardaic surgery or cardiac catheterisation. It can also be observed in different clinical settings as infectious diseases, neoplasia, and inflammatory diseases. It has a wide range of presentation and in some settings it can appear a dramatic event. Although a rare finding during acute rheumatic fever, with a transient course, it may need a specific and intensive treatment. We report a case of complete atrio-ventricular (AV) block in whom an Adams-Stokes attack was the first symptom of acute rheumatic carditis. We also reviewed the literature on complete atrio-ventricular block in acute rheumatic fever.

## Case report

A 14-year-old Italian boy, weight 50 kg, was admitted to the emergency room of our Paediatric Department for syncope which occurred at home after he got out of bed. He had complained of transient thoracic pain the day before. On admission, the patient appeared extremely pale. Severe bradycardia (30 beats/minute) was found, blood pressure was 115/65 mmHg, respiratory rate 24/minute and transcutaneous oxygen saturation was 98%. A grade 2/6 systolic murmur was audible at the apex. The remaining physical examination was unremarkable.

**Figure 1:**
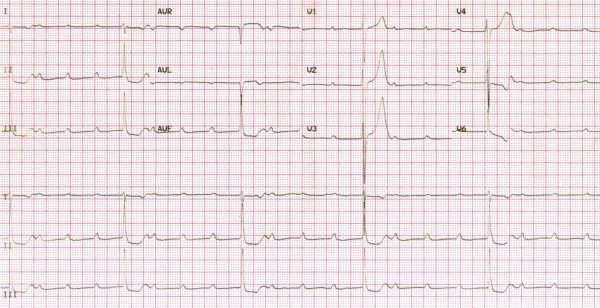
**Electrocardiogram showing complete A-V block with a ventricular rate of 30 bpm** .

**Figure 2:**
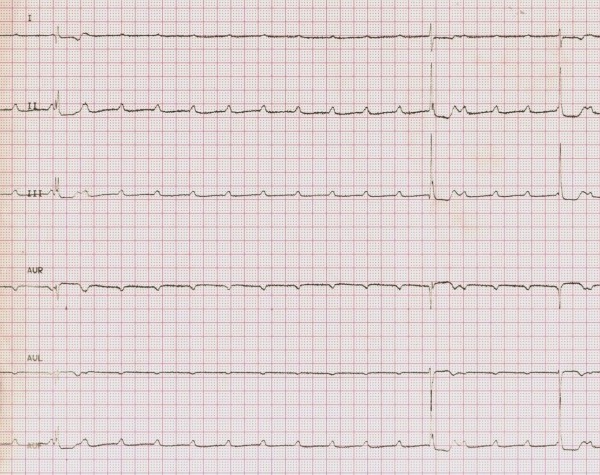
**Electrocardiogram showing paroxysmal AV block and a 5.52 second period of asystolia** .

The ECG showed a complete AV block with narrow QRS and a ventricular rate of 30 beats/minute (Figure 1). A 5.52 second period of asystole was recorded as well (Figure 2). Transthoracic echocardiography revealed mild mitral regurgitation, no cardiac chamber enlargement (left ventricle end-diastolic diameter was 47 mm) and normal contractility (ejection fraction 67%, shortening fraction 37%); a temporary pacemaker was implanted via the right femoral vein. In the suspicion of an inflammatory etiology, intravenous methyl-prednisolone (20 mg b.i.d) was started.

History pointed out a febrile pharyngitis occurred about one month before. At that moment, a rapid antigen detection test was positive for β-haemolytic group A Streptococcus. Amoxicillin plus clavulanate, 1 gram b.i.d, had been prescribed for ten days.

Laboratory investigations revealed neutrophilic leukocytosis (WBC 17.750/mm^3^, N 82%), elevation of ESR and CRP (72 mm/hr and 136 mg/L, respectively), elevated streptococcal antibodies (ASO titre 3.220 U/mL, streptozyme test positive 1/5000, anti-streptokinase antibodies positive 1/2560). The throat culture for β-haemolytic group A Streptococcus was negative. Myocardial necrosis indices and Borrelia Burgdorferi antibodies also were negative.

After 24 hours, the patient recovered sinus rhythm (HR = 80 beats/minute) with first degree AV block (PR duration 250 milliseconds). A second echocardiography confirmed the mild mitral regurgitation, but also showed a slight thickening of the aortic leaflets with trivial aortic regurgitation. The temporary pacemaker was removed and the anti-inflammatory treatment was continued with oral prednisone 25 mg b.i.d. for two weeks. When the normalisation of the inflammatory indices was achieved, steroid treatment was progressively tapered and acetylsalicylic acid 750 mg q.i.d. was started and continued for four weeks. ECG performed on fourth day after admission showed a normal sinus rhythm with a normal PR interval duration. Forty days after the first examination, echocardiography showed complete resolution of both mitral and aortic regurgitation; the Holter ECG showed a sinus rhythm with normal AV conduction.

The final diagnosis was Adams-Stokes attack due to complete AV block in the course of acute rheumatic carditis.

## Discussion

The most common cause of acquired complete AV block in the paediatric age group is direct injury to conduction tissue during cardiac surgery or cardiac catheterisation. In addition, complete atrio-ventricular block can be observed in infectious diseases as viral myocarditis, diphtheria, Lyme disease, in inflammatory illnesses such as acute rheumatic fever, metabolic diseases as Kearns-Sayre syndrome, drug toxicity (digoxin, beta-blockers, calcium-channel blockers), Chagas disease, tuberous sclerosis, intra-cardiac tumours, ischemia during coronary events or after mediastinal radiation.

The most common AV conduction abnormality found during acute rheumatic fever is first degree AV block, which was recognised in 72.5% of the Clarke’s series and in 72.3% of Zalzstein’s series (1, 2). Second degree AV block of Mobitz type I is much less frequent (2.6% in Clarke’s and 1.5% in Zalzstein’s series). Complete AV block was diagnosed in 0.6% of the Clarke’s and in 4.6% of Zalzstein’s series. Other types of rhythm abnormalities recognised during acute rheumatic fever include sinus node dysfunction, junctional rhythm and junctional tachycardia, ventricular tachycardia, torsade de pointes due to QT interval prolongation and complete left bundle branch block.

In Clarke’s series, only one of the three patients with complete AV block presented with an Adams-Stokes attack (1). All three patients with complete AV block of Zalzstein’s series were asymptomatic (2).

In our case, the Adams-Stokes attack was the first symptom of acute rheumatic fever. This occurred because of the high degree of complete AV block, with periods of asystole longer than five seconds.

We examined the literature in order to collect other cases of complete AV block due to rheumatic fever. We looked through PubMed’s MeSH vocabulary by inserting “rheumatic fever”, “atrio-ventricular block”, and “Adams-Stokes attack”.

We were able to find 19 full-text papers in which 25 cases of complete AV block due to rheumatic fever were reported [1-19]. Ten of the 25 patients experienced an Adams-Stokes attack [1,3,5,6,9,11-15] (Table 1).

**Table 1:** Cases of complete atrio-ventricular block in acute rheumatic fever collected from the literature

**Author**	**Age (years), gender**	**Adams-Stokes attack**	**Degree of AV block**	**Pacing**	**Duration of complete AV block**
**(Ref number)**					
Arcuri [3]	47, m	Yes	Intermittent complete AV block	no	7 days
Barold [4]	39, m	No	From I to III	no	5 days
Baracchi [5]	33, m	No	III	No	4 days
	13, m	Yes	From II for 10 days to III	no	3 days
Clarke [1]	paediatric	Yes	From I to III	yes	8 days
	paediatric	No	From I to III	no	unknown
	paediatric	No	From I to III	no	unknown
Duran [6]	17, f	Yes	From III to II	yes	5 days
Filberbraum [16]	unknown	unknown	III	unknown	unknown
Guven [7]	9, m	No	From II to III	no	no improvement in rhythm at the 3^rd^ month
Hee Yoo [8]	13, m	No	From III to II	no	3 days
Lenox [9]	13, m	Yes	III	yes	unknown
Malik [10]	16, m	No	From I to III	no	a few minutes
Mohindra [11]	38, m	Yes	III	yes	unknown
Montano [17]	9, f	No	III	no	10 days
Poberezovskii [12]	paediatric	Yes	III	unknown	unknown
Rojas [13]	15, unknown	Yes	III	yes	4 days
Shah [18]	12, f	unknown	III	unknown	unknown
Stocker [19]	paediatric	unknown	III	unknown	unknown
	paediatric	unknown	III	unknown	unknown
Tampieri [14]	37, m	Yes	III	yes	2 days
Thomas [15]	12, m	Yes	III	yes	36 hours
Zalzestein [2]	3 patients range 9 to 11 (1 m, 2 f)	No	III	No	from 30 to 48 hours
		No	III	No	
		No	III	No	

Nineteen of the 25 patients with complete AV block were certainly in the paediatric age group [1,2,5,9,10,12,15,18,19]. Seven of the 19 experienced an Adams-Stokes attack [1,5,6,9,12,15].

In 16 out of 25 cases, the duration of the AV block was reported: it lasted from a few minutes to ten days [1,2,4-8,10,15]; in one case, an ECG three months later showed persistence of the complete block [7]. Pacemaker implantation was necessary in seven cases.

## Conclusions

Complete heart block during acute rheumatic fever is rare. Despite it can appear as a dramatic event, it is usually transient, resolving in few days after initiating anti-inflammatory treatment. Specific treatment, such as insertion of a temporary pacemaker, should be considered only when complete AV block leads to an Adams-Stokes attack. In our patient, the Adams-Stokes attack was the first symptom of rheumatic fever. Rheumatic fever must be kept in mind in the diagnostic work-up of patients with acquired complete AV block, particularly when it occurs in paediatric patients.

Written informed consent has been obtained from the parents of the patient for publication of this case report and any accompanying images.

## Abbreviations

ASO: Antibodies to streptolysin O; AV: Atrio – ventricular; CRP: C-reactive proteine; ECG: Electrocardigraphy; ESR: Erytrocyte sedimentation rate; WBC: White blood cells.

## Competing interests

The authors declare that they have no competing interest.

## Authors’ contributions

NC: Data analysis, data interpretation and writing. IB: Literature search and writing. BT: Literature search, figures. EV: Data collection. SF: Data collection. AA: Writing. All authors read and approved the final manuscript.
